# Associations of bedtime, sleep duration, and sleep quality with semen quality in males seeking fertility treatment: a preliminary study

**DOI:** 10.1186/s12610-020-00103-7

**Published:** 2020-04-23

**Authors:** Julius Edward Miller Hvidt, Ulla Breth Knudsen, Robert Zachariae, Hans Jakob Ingerslev, Marie Tholstrup Philipsen, Yoon Frederiksen

**Affiliations:** 1 Department of Clinical Medicine, Aarhus University, 8000 Aarhus, Denmark; 2 Horsens Fertility Clinic, Horsens Regional Hospital, 8700 Horsens, Denmark; 3 Department of Psychology and Behavioural Sciences, Aarhus University, 8000 Aarhus, Denmark; 4 Department of Oncology, Aarhus University Hospital, 8000 Aarhus, Denmark; 5 Fertility Unit, Aalborg University Hospital, Søndre Skovvej 3, 9000 Aalborg, Denmark; 6 Department of Obstetrics and Gynaecology, Aarhus University Hospital, Palle Juul-Jensens Boulevard 99, 8200 Aarhus N, Denmark; 7 The Unit for Clinical Sexology, the Department of Affective Disorders, Aarhus University Hospital, Psychiatry, Palle-Juul Jensens Boulevard 175, 8200 Aarhus N, Denmark

**Keywords:** Sommeil, Heure du Coucher, Durée du Sommeil, Qualité du Sommeil, Qualité du Sperme, Fertilité masculine, Sleep, Bedtime, sleep duration, sleep quality, semen quality, Male fertility

## Abstract

**Background:**

Poor sleep has been linked to a number of adverse health outcomes. Recent studies suggest that late bedtimes, short or long sleep durations, and poor sleep quality may impair semen quality. No study has previously explored all three factors in relation to semen quality.

**Results:**

One hundred and four men and their partners treated at three fertility clinics in Denmark between 2010 and 2012 completed an online-version of the Pittsburgh Sleep Quality Index (PSQI). The results of the semen analyses conducted at the fertility clinics were self-reported and categorised as normal or reduced.

Early bedtime (< 10:30 PM) was more often associated with normal semen quality compared with both regular (10:30 PM-11:29 PM) and late (≥11:30 PM) bedtime (OR: 2.75, 95%CI: 1.1–7.1, *p* = 0.04 and OR: 3.97, 95%CI: 1.2–13.5, *p* = 0.03). Conventional sleep duration (7.5–7.99 h) was more often associated with normal semen quality than both short (7.0–7.49 h) and very short (< 7.0 h) sleep duration (OR: 1.36, 95% CI: 1.2–12.9, *p* = 0.03 and OR: 6.18, 95%CI: 1.6–24.2, *p* = 0.01). Although poor sleep quality was associated with reduced semen quality in the descriptive statistics (*p* = 0.04), no differences were found between optimal (PSQI ≤6) and either borderline (PSQI 7–8) or poor (PSQI ≥9) sleep quality (OR: 1.19, 95%CI: 0.4–3.4, *p* = 0.75 and OR: 2.43, 95%CI: 0.8–7.1, *p* = 0.11) in multivariate regression models.

**Conclusion:**

Early bedtimes (< 10:30 PM) and conventional sleep duration (7.5–7.99 h) were associated with self-reported normal semen quality. The role of subjective sleep quality remains uncertain.

## Abstract (fr)

**Contexte:**

Un mauvais sommeil a été associé à plusieurs issues néfastes pour la santé. De récentes études suggèrent que les heures de coucher tardives, des durées de sommeil courtes ou longues, et une mauvaise qualité de sommeil altèrent la qualité du sperme. Aucune étude n’a à ce jour exploré les trois facteurs en relation avec la qualité du sperme.

**Résultats:**

Cent quatre hommes et leurs partenaires traités dans trois cliniques de fertilité au Danemark entre 2010 et 2012 ont rempli un questionnaire correspondant à une version en ligne de l’Indice de Qualité du Sommeil de Pittsburg (IQSP). Les résultats des analyses de sperme réalisées dans les cliniques de fertilité ont été autodéclarés et classés comme normaux ou réduits.

Une heure de coucher précoce (< 22 h30) était plus souvent associée à une qualité normale du sperme comparativement à la fois à une heure régulière (22 h30-23 h29) et à une heure tardive (≥23 h30) de coucher (OR: 2.75, 95% CI: 1.1–7.1, *p* = 0.04 et OR: 3.97, 95% CI: 1.2–13.5, *p* = 0.03). Une durée conventionnelle de sommeil (7.5–7.99 heures) était plus souvent associée à une qualité normale du sperme qu’une courte (7.0–7.49 heures) et qu’une très courte (< 7.0 heures) durée de sommeil (OR: 1.36, 95% CI: 1.2–12.9, p = 0.03 et OR: 6.18, 95% CI: 1.6–24.2, *p* = 0.01). Bien qu’une mauvaise qualité du sommeil ait été associée à une qualité réduite du sperme dans les statistiques descriptives (*p* = 0.04), aucune différence n’a été retrouvée entre une qualité du sommeil optimale (IQSP ≤6) et une qualité soit limite (IQSP 7–8) ou soit pauvre (IQSP ≥9) du sommeil (OR: 1.19, 95% CI: 0.4–3.4, *p* = 0.75 et OR: 2.43, 95% CI: 0.8–7.1, *p* = 0.11) dans les modèles de régression multivariée.

**Conclusions:**

Des heures de coucher précoces (< 22 h30) et une durée conventionnelle de sommeil (7.5–7.99 heures) ont été associées à une qualité normale autodéclarée du sperme. Un éventuel rôle de la qualité suggestive du sommeil reste incertain.

## Background

A growing number of studies indicate an association between poor sleep and negative health outcomes. These include increased risk of cardio-metabolic complications, hypertension, obesity, type 2-diabetes, cardiovascular disease, urologic complications, cancer, and depression [1–6]. Results from a limited number of recent studies suggest that inadequate bedtimes, short sleep duration, and poor sleep, assessed as poor self-reported sleep quality, may impact semen quality [7–13].

Only one study by Liu et al. [8] has directly examined bedtime in relation to semen quality in a study of 981 healthy Chinese men. The study found that late bedtime and short or long sleep durations negatively impacted semen quality. The combination of a short sleep duration and a late bedtime seemed to further decrease semen quality. A recent study by Green et al. [14] on 116 men undergoing fertility evaluation also showed that bedtime usage of smartphones and tablets was negatively associated with semen quality.

Sleep duration itself has been linked to semen quality in fertile [9] and in infertile [13, 14] men. Chen et al. [9] found the optimal sleep duration to be 7.0–7.5 h in 796 Chinese fertile men. Green et al. [14] found a positive correlation between sleep duration and total sperm and motility. Shi et al. [13] found sperm concentrations to be lower in men with a short sleep duration and higher in men with a long sleep duration in 328 Hong Kongese men.

To the best of our knowledge, only three studies have used validated self-report questionnaires or subscales to measure sleep quality in association with semen quality. Chen et al. [9] found an association between Pittsburgh Sleep Quality Index (PSQI) global scores [15] and semen parameters (volume and total sperm number) in a study of 656 fertile Chinese men. Jensen et al. [10] found an inverse u-shaped relationship between sleep quality and semen quality using a modified 4-item version of the Karolinska Sleep Questionnaire [16] in a sample of 953 fertile Danish men. Green et al. [14] used the PSQI and the Karolinska Sleepiness Scale (KSS) and found that subjective sleepiness measured on the KSS was associated with reduced motility and sperm count. The study did not report any correlations between global PSQI scores and semen quality.

Poor sleep classified more loosely as difficulties falling asleep and lying awake at night has also been associated with reduced sperm volume and motility in 382 Italian men seeking fertility treatment in a study by Viganò et al. [7].

Overall, only little is known about associations between sleep quality and semen quality in men, whether fertile or infertile. So far, only three studies have used validated instruments to measure sleep quality, and no studies have investigated bedtime, sleep duration, and sleep quality in the same group of men in relation to semen quality. The present study addressed this knowledge gap by exploring associations between semen quality and sleep in men from couples undergoing fertility treatment.

## Methods

The data used in the present study was collected as part of a previously reported randomized controlled trial (RCT) [17]. The primary objective of that study was to evaluate the effects of a home-based expressive writing intervention on distress and pregnancy outcomes in couples receiving treatment with assisted reproductive technology (ART).

### Participants and selection criteria

Data was collected from couples scheduled to receive either in vitro fertilization (IVF) or intracytoplasmic sperm injection (ICSI) treatments at three Danish fertility clinics. The participating fertility clinics were: The Fertility Clinic at Aarhus University Hospital, Skive Fertility Clinic, and Brædstrup Fertility Clinic. Data was collected between November 2010 and July 2012.

The inclusion criteria of the original study were: scheduled IVF or ICSI treatment, age between 18 and 45 years, and ability to write and read Danish. For further information on participant characteristics, please see Table 1 and [17]. Exclusion criteria in the original study were: women without a partner and couples assigned to preimplantation genetic testing, as they tended to follow a different treatment path.

**Table 1:** Demographic and sleep characteristics of study population presented as means and standard deviations

Demographic	Total	Reduced semen quality		***P***-value
Mean (SD)	(*N* = 104)	No (*N* = 48)	Yes (*N* = 56)	
Age (years)	34.00 (5.5)	33.67 (5.1)	34.30 (5.8)	0.55
Education ^a^	3.57 (1.2)	3.77 (1.1)	3.39 (1.3)	0.10
Income ^b^	3.90 (1.0)	4.06 (0.9)	3.77 (1.0)	0.12
Tobacco smoking ^c^	0.06 (0.2)	0.04 (0.2)	0.07 (0.3)	0.51
Alcohol consumption (standard drinks/week)	6.86 (8.5)	7.13 (10.1)	6.63 (6.8)	0.77
Bedtime (hour in 24-h format)	22.72 (0.9)	22.58 (0.8)	22.86 (0.9)	0.09
Sleep duration (hours/day)	7.24 (0.9)	7.42 (0.66)	7.09 (1.0)	0.05*
Global PSQI score	8.17 (2.3)	7.69 (1.9)	8.59 (2.4)	0.04*

^a^ (1) Unskilled – no further education after high school, (2) basic education, (3) technical education (e.g. hairdresser, carpenter, mechanic, chef, plumber), (4) professional Bachelor’s degree (e.g. nurse, teacher, social worker), and (5) Master’s degree at university level. ^b^ Household annual income of: (1) < 200.000 DKK, (2) 200.000–350.000 DKK, (3) 351.000–500.000 DKK, (4) 501.000–700.000 DKK, (5) > 700.000 DKK. ^c^ % of total with (0) not currently smoking, (1) currently smoking. SD: Standard deviation. N: Number of observations. PSQI: Pittsburgh Sleep Quality Index global score. *P*-value based on simple t-tests assuming unequal variance. * Significant at *p* < 0.05

**Fig. 1:**
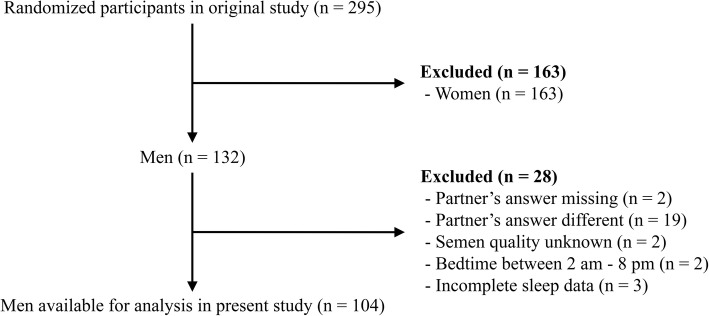
Flowchart illustrating the participant selection. Flowchart descriping participant selection in the present study

A total of 295 participants (163 women and 132 men) were included in the original study [17]. Of these, 28 men were excluded from the present study due to a) discrepancy between the answers given by the men and their partners regarding male semen quality, b) missing data, or c) the bedtime reported not to be during the evening (8 pm - 2 am). This resulted in a total of 104 men eligible for further analysis (see Fig. 1). For further details, see [17].

### Procedure and study design

Couples received oral and written information about the research project at a scheduled standard consultation at the fertility clinics prior to entering the project. The study protocol was approved by the Danish Data Protection Agency as well as the Central Danish Region Committees on Health Research Ethics (ref. number M-2010015). The study was carried out according to the Declaration of Helsinki Principles and registered at: www.clinicaltrials.gov as NCT01187095. Consenting participants received an email with links to online-questionnaires (SurveyXact; Rambøll) at baseline (t1), post-intervention (t2), and follow-up (t3). The present study only focused on the association between sleep characteristics and self-reported semen quality at baseline (t1). Semen quality was measured at the fertility clinics by trained medical staff in accordance with the specification of the World Health Organisation (WHO) within 3 months prior to inclusion, and the conclusion of the analysis was self-reported by the participants as “normal” or “reduced”.

### Measures

Sociodemographic and medical information was obtained from questionnaires and medical records. The present study focused on the self-reported: cause of infertility, sleep quality, sleep duration, and bedtime. For further information on the remaining measures included in the study, please see to Frederiksen et al. [17].

#### Cause of infertility

All participants reported the cause of infertility in the questionnaire. Men were included in the present study only if both partners gave identical answers in relation to semen quality. Individual semen parameters from the same man may vary from sample to sample [18]. The clinical diagnosis “reduced semen quality” was routinely based on at least two semen samples. Therefore, we chose a broad dichotomization into “normal” or “reduced” semen quality. It should be noted that it was not possible to evaluate the results of the original semen analysis directly as civil registration numbers had been deleted from the data of the original study due to data protection laws.

#### Bedtimes

The PSQI sub-component ‘bedtime’ was also investigated separately. Existing literature on semen quality and bedtime has categorized bedtimes as before 10 PM, 10 PM to 12 AM, and after 12 AM [8]. A similar categorization was not feasible in the present study due to relatively few data points. Average bedtime for Danish men have been reported to be around 11 PM [19]. We therefore chose 11 PM as the “normal” bedtime including intervals of 30 min pre and post 11 PM. This resulted in the following three bedtime categories: early (8:00 PM - 10:29 PM), regular (10:30 PM - 11:29 PM), and late (11:30 PM - 1:59 AM).

#### Sleep duration

The PSQI sub-component ‘sleep duration’ was explored separately, following a similar approach to Chen et al. [9] using 30-min intervals. The number of intervals was fewer in the present study due to data limitations. A sleep duration of 7.5–7.99 h was used as reference, as the average sleep duration for Danish men aged 30–45 years was 7.8 h [20]. The sleep duration groups in the present study were: very short (< 7 h), short (7.0–7.49 h), conventional (7.5–7.99 h), and long (≥8.0 h).

#### Sleep quality

Sleep quality was assessed with the Pittsburgh Sleep Quality Index (PSQI) global scores [15]. The PSQI contains 19 items evaluating sleep quality over a one-month period and yields seven component scores and a global sleep quality score. Global sleep quality scores range from 0 to 21 with higher scores indicating poorer sleep quality. The PSQI components include subjective sleep quality, sleep duration, sleep efficiency, use of sleep medication, sleep onset latency, and daytime dysfunction. Global PSQI sleep quality scores were categorized as optimal (PSQI ≤6), borderline (PSQI 7–8), and poor (PSQI ≥9) as previously suggested [21].

### Statistical analysis

Previous research suggested a non-linear inverse u-shaped association between semen quality and both sleep duration and sleep quality. Bedtime, sleep duration, and global PSQI scores were therefore recoded into categorical variables to investigate how each parameter was associated with reduced semen quality.

Descriptive statistics with means and standard deviations are reported in both an aggregated and a subdivided version with respect to reduced and non-reduced semen quality. A simple t-test not assuming equal variances was used to test differences between means. Sleep related variables are presented and used in their continuous forms instead of the categorical forms used in the subsequent regression models of the paper.

The association between semen quality and sleep parameters was analyzed in binary logistic regression models. Both unadjusted and adjusted regression models were created. Adjusted regression models were adjusted for: age, alcohol consumption (number of standard drinks each week), and current smoking status (smoker/non-smoker).

Assumptions regarding logistic regression modelling were met with no variable inflation factor exceeding 2.5. Data analysis was conducted with IBM SPSS version 22 using 5% as the statistical significance level.

## Results

### Participant characteristics

Demographic and sleep characteristics of the 104 men included in the final analysis are shown in Table 1. The participants had an average bedtime of 10:42 PM, an average sleep duration of 7 h and 12 min, and an average global PSQI score of 8.2. Men with self-reported reduced semen quality had a significantly shorter sleep duration (*p* = 0.05) and poorer sleep quality (global PSQI) (*p* = 0.04) than men with normal semen quality. There were no statistically significant differences between men with reduced and normal semen quality with respect to bedtime, age, educational level, income, smoking status or alcohol consumption.

### Associations of sleep parameters with semen quality in regression models

#### Bedtime and semen quality

**Fig. 2:**
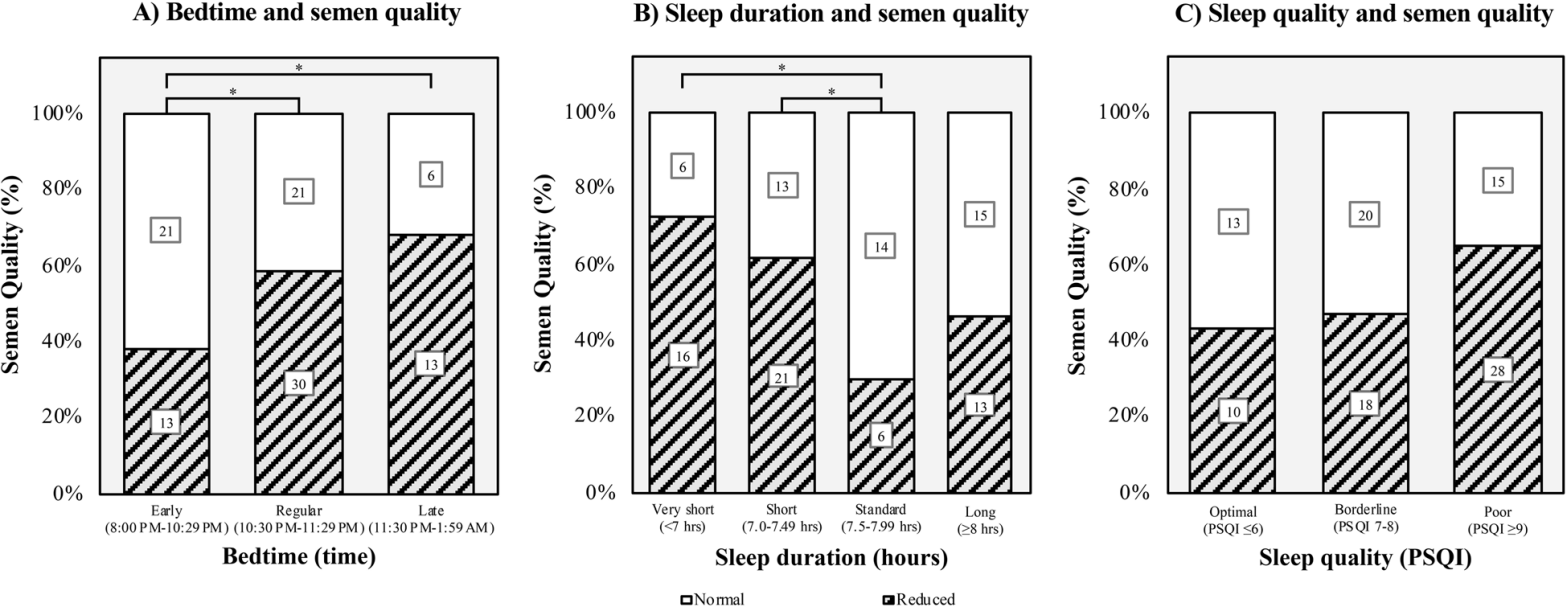
Proportions of normal and reduced semen quality in relation to sleep parameters. **a** Proportions of normal and reduced semen quality grouped by early (8:00 PM - 10:29 PM), regular (10:30 PM - 11:29 PM), and late (11:30 PM - 1:59 AM) bedtime. **b** Proportions of normal and reduced semen quality grouped by very short (< 7 h), short (7–7.49 h), conventional (7.5–7.99 h), and long (≥8 h) sleep duration. **C)** Proportions of normal and reduced semen quality grouped by optimal (PSQI ≤6), borderline (PSQI 7–8), and poor (PSQI ≥9) sleep quality. The numbers in the bars represent number of observations. PSQI: Pittsburgh Sleep Quality Index global score. *Statistically significant at *p* < 0.05 when adjusting for age, smoking status, and alcohol consumption

Both the unadjusted and adjusted regression models showed a significant association between later bedtimes and reduced semen quality (see Table 2). In the unadjusted model, early bedtime was more often associated with normal semen quality compared to late bedtime (OR: 3.5, 95% CI: 1.1–11.5, *p* = 0.04). However, there was no difference between early vs. regular bedtime (OR: 2.31, 95% CI: 0.95–5.6, *p* = 0.07). In the model adjusted for age, smoking status, and alcohol consumption, there was a difference between early vs. regular (OR: 1.01, 95% CI: 1.1–7.1, p = 0.04) and late bedtime (OR: 3.97, 95% CI: 1.2–13.5, *p* = 0.03) with early bedtime more often associated with normal semen quality (see Fig. 2-a).

**Table 2:** Association between sleep parameters and reduced semen quality

Variable	Unadjusted			Adjusted		
	B (SE)	Odds Ratio (95% CI)	*P*-value	B (SE)	Odds Ratio (95% CI)	*P*-value
**Model 1 (Bedtime)**						
Early Bedtime (8:00 PM - 10:29 PM)	Reference	1.00 (reference)		Reference	1.00 (reference)	
Regular Bedtime (10:30 PM - 11:29 PM)	0.84 (0.45)	2.31 (0.95, 5.6)	0.07	**1.01 (0.48)**	**2.75 (1.1, 7.1)**	0.04*
Late Bedtime (11:30 PM - 1:59 AM)	**1.25 (0.61)**	**3.50 (1.1, 11.5)**	0.04*	**1.38 (0.62)**	**3.97 (1.2, 13.5)**	0.03*
**Model 2 (Sleep duration)**						
Very Short Sleep Duratio (< 7 h)	**1.83 (0.68)**	**6.22 (1.6, 23.8)**	0.01*	**1.82 (0.70)**	**6.18 (1.6, 24.2)**	0.01*
Short Sleep Duration (7.0–7.49 h)	**1.33 (0.60)**	**3.77 (1.2, 12.3)**	0.03*	**1.36 (0.61)**	**3.88 (1.2, 12.9)**	0.03*
Conventional Sleep Duration (7.5–7.9 h)	Reference	1.00 (reference)		Reference	1.00 (reference)	
Long Sleep Duration (≥8.0 h)	0.70 (0.62)	2.02 (0.6, 6.8)	0.25	0.75 (0.65)	2.11 (0.6, 7.5)	0.25
**Model 3 (Sleep Quality)**						
Optimal Sleep Quality (PSQI ≤6)	Reference	1.00 (reference)		Reference	1.00 (reference)	
Borderline Sleep Quality (PSQI 7–8)	0.16 (0.53)	1.2 (0.4, 3.3)	0.77	0.17 (0.54)	1.19 (0.4, 3.4)	0.75
Poor Sleep Quality (PSQI ≥9)	0.89 (0.53)	2.43 (0.9, 6.8)	0.09	0.89 (0.55)	2.43 (0.8, 7.1)	0.11

Calculations are based on binary logistic regressions modelling the association between sleep parameters and semen quality in relation to the reference group. Odds ratio: odds of reporting reduced semen quality. Model 1: investigates association between bedtimes and semen quality. Model 2: investigates association between sleep durations and semen quality. Model 3: investigates association between sleep quality and semen quality. Adjusted models include age, smoking status, and alcohol consumption. B: beta-coefficient, SE: standard error, 95% CI: 95% confidence interval. *PSQI* Pittsburgh Sleep Quality Index global score. *Significant at *p* < 0.05

#### Sleep duration and semen quality

Both the unadjusted and adjusted binary regression models revealed statistically significant associations between shorter sleep durations and reduced semen quality (see Table 2). The unadjusted models showed significant differences between both conventional vs. very short (OR: 6.22, 95% CI: 1.6–23.8, *p* = 0.01) and short sleep duration (OR: 3.77, 95% CI: 1.2–12.3, *p* = 0.03). No difference was found between conventional and long sleep duration (OR: 2.02, 95% CI: 0.6–6.8, *p* = 0.25). The models adjusted for age, smoking status, and alcohol consumption remained statistically significant for both conventional vs. very short (OR: 6.18, 95% CI: 1.6–24.2, p = 0.01) and short sleep duration (OR: 1.36, 95% CI: 1.2–12.9, p = 0.03), and no difference was found between conventional and long sleep duration (OR: 0.75, 95% CI: 0.6–7.5, p = 0.25) (see Fig. 2-b).

#### Sleep quality and semen quality

No association was found between sleep quality measured as global PSQI scores and semen quality in either the unadjusted or adjusted regression models (see Table 2). The unadjusted binary logistic regression model showed no difference between optimal vs. borderline (OR: 1.2, 95% CI: 0.4–3.3, *p* = 0.77) or poor sleep quality (OR: 2.43, 95% CI: 0.9–6.8, *p* = 0.09). Similar results were found when adjusting for age, smoking status, and alcohol consumption for both optimal vs. borderline (OR: 1.19, 95% CI: 0.4–3.4, *p* = 0.75) and poor sleep quality (OR: 2.43, 95% CI: 0.8–7.1, *p* = 0.11) (see Fig. 2-c).

## Discussion

To our knowledge, this is the first study to examine the association of the three parameters of bedtime, sleep duration, and sleep quality with semen quality in men seeking treatment suspected of infertility. The results suggested that early bedtime (< 10:30 PM) and conventional sleep duration (7.5–7.99 h) were both associated with self-reported normal semen quality. The role of sleep quality, however, remains uncertain, and the number of participants limits the generalizability of the results.

### Bedtime

In the present study, late bedtime was associated with reduced semen quality in men suspected of infertility. This is in agreement with Liu et al. [8], who conducted a study with men not suspected of infertility. In our study, sleep duration and bedtime were significantly correlated with later bedtimes associated with shorter sleep durations. This is in agreement with other studies which have found that the average bedtime predicted sleep duration [19]. In an unadjusted regression model with both bedtime and sleep duration, bedtime became insignificant indicating that sleep duration is more strongly associated with semen quality than bedtime in our study (see Supplementary material, Table S1).

One experimental study has shown that a short sleep duration coupled with a late bedtime increased the negative effect on semen quality compared to having a short sleep duration and a regular or early bedtime [8]. This was done by randomly assigning participants a sleep duration and a bedtime. These findings could be related to circadian disruption, which has been linked to dysregulation of gene transcription of the Circadian Locomotor Output Cycles Kaput (CLOCK) genes [22] in which single nucleotide polymorphisms have been linked to reduced semen volume, sperm count, and sperm motility [23]. The disruption of the circadian rhythm has also been associated with lower levels of urinary 8-hydroxydeoxyguanosine likely caused by reduced melatonin levels indicating a reduced ability to repair oxidative deoxyribonucleic acid (DNA) damage which is important for fertility [24]. In line with this, Shi et al. [13] found a higher DNA fragmentation index (DFI) in men with irregular sleep. Prescribing regiments of reasonable bedtimes with relevant sleep durations could prove an important factor in increasing the fertility of men based on the currently available literature and the results of the present study.

### Sleep duration

In the present study, a sleep duration shorter than 7.5 h was associated with reduced semen quality. Shorter sleep durations have previously been linked to reduced semen quality in a limited number of studies [8, 9, 13, 14]. The optimal sleep duration in relation to semen quality in the most comparable study, which was conducted with Chinese university students, was 7.0–7.5 h [9] and thus shorter than in our study. While we have no clear explanation for the discrepancy, it could be due to both age and cultural differences [25]. The average sleep duration is thus 7.8 h for Danish men between the ages of 30–45 years [20] and the average sleep duration of Chinese university students is 7.08 h [26].

In the present study, we did not find an association between a longer sleep duration of more than 8 h and semen quality. This could be due to the small number of participants with a long sleep duration resulting in insufficient statistical power. A sleep duration of 8–9 h has not been reported to reduce semen quality, but sleeping longer than 9 h has been associated with reduced semen quality in two studies with fertile men [8, 9]. However, sleeping longer than 8 h has also been associated with better semen quality in men suspected of infertility [13, 14]. This could be due to methodological differences between the studies. The impact of a long sleep duration on semen quality requires further investigation to determine if longer sleep duration can benefit men with reduced fertility.

Optimizing sleep duration of men could prove a relevant factor in fertility treatment. One study has shown that participants who by themselves changed their sleep duration towards the local convention, subsequently reported better semen quality than those who did not [9]. The association between optimal sleep duration and good semen quality could be due to lower levels of anti-sperm antibodies (ASA), which have been found elevated in individuals with restricted sleep duration [8].

### Sleep quality

No significant association was observed in this study between global PSQI scores and semen quality in the regression models. This could be due to the limited sample size (*N* = 104) as the difference between optimal vs. poor sleep quality almost reached significance in the unadjusted model (OR: 2.43, 95% CI: 0.9–6.8, *p* = 0.09) and explains why the simple t-test in continuous data reached statistical significance (*p* = 0.04). Two other studies have found an association using either global PSQI scores (*N* = 656) [9] or a modified 4-item Karolinska Sleep Questionnaire (*N* = 953) [10]. However, it should be noted that the study utilizing the global PSQI score found that *“… adjusting for PSQI conferred little difference on the association between sleep duration and semen parameters, whereas PSQI become insignificant in the regression models”* [9]. In conjunction with this previous finding, the results of the present study could indicate that sleep quality may be associated with semen quality.

### Strengths and limitations

This is the first study to investigate both bedtime and sleep duration in relation to semen quality in men suspected of infertility. Furthermore, it is the first study to examine the association between the three parameters: bedtime, sleep duration, and sleep quality in relation to semen quality in the same group of men. It is one of very few studies to utilize a thoroughly validated sleep quality instrument like the PSQI in relation to research on the fertility of men.

A number of limitations should be noted. First, the present study is cross-sectional and based on self-reported sleep quality and fertility data. This implies that no causation can be drawn from this study. Second, the limited response rate (23%) may increase the risk of response bias, and the relatively small number of men included in the study may reduce the statistical power, increase the risk of type-2 errors, and limit the generalizability of the results. Third, of particular concern could be that semen quality was self-reported, which could introduce increased risk of erroneous reporting, miscommunication, misremembered information, and reporting bias. It was not possible to evaluate the data directly in the participants’ hospital journals due to civil registration numbers having been deleted due to data protection considerations. This present paper took steps towards reducing these limitations by, for example, only including men from couples that provided the same answer regarding semen quality (see Fig. 1). The dichotomization of semen quality into “normal” and “reduced” reduces the information provided in the data. However, it is well known that semen parameters taken from samples from the same man may vary considerably [27], hence, the dichotomization can be interpreted as representing a relatively more robust parameter [18]. Fourth, semen quality is associated with a range of lifestyle factors [28], many of which our study was unable to adjust for. Fifth, sleep data was also self-reported rather than measured objectively, e.g., using polysomnography or actigraphy introducing potential errors. Hence, a potential risk of confounding exists within our models due to data limitations and it should be noted that no causality can be made from this study due to the study design.

## Conclusion

This is the first study to examine all three factors of bedtime, sleep duration, and sleep quality in terms of global PSQI scores in the same group of men seeking fertility treatment. The results indicated that short sleep duration and late bedtime were statistically significantly associated with reduced semen quality. Although the unadjusted models showed that poor sleep quality was assosicated with reduced semen quality (*p* = 0.04), the association did not reach statistical significance when investigated with a multivariate model, adjusting for other relevant factors. Thus, the results of the present study provide further support for previous findings suggesting that sleep plays a role in male fertility.

## Supplementary information

**Additional file 1: Table S1.** Association between bedtime and sleep duration with reduced semen quality.

## Data Availability

The datasets used and analyzed during the current study are available from the coresponding author on reasonable request.

## Abbreviations

ASA: Anti-sperm antibodies
CLOCK: Circadian Locomotor Output Cycles Kaput
DNA: Deoxyribonucleic Acid
ICSI: Intracytoplasmic sperm injection
IVF: In vitro fertilization
KSS: Karolinska Sleepiness Scale
PSQI: Pittsburgh Sleep Quality Index
WHO: World Health Organisation
