# Supplementary material for: Associations of bedtime, sleep duration, and sleep quality with semen quality in males seeking fertility treatment: a preliminary study
Source: Basic Clin Androl. 2020 Apr 23;30:5. doi: 10.1186/s12610-020-00103-7 (PMC7181488; doi:10.1186/s12610-020-00103-7)
Supplement: Supplementary file 1 — Additional file 1: Table S1. Association between bedtime and sleep duration with reduced semen quality. [file 12610_2020_103_MOESM1_ESM.docx]

# Supplementary material

**Table S1**: Association between bedtime and sleep duration with reduced semen quality

|  | **Unadjusted** | | |
| --- | --- | --- | --- |
|  |  |  |  |
| **Variable** | **B (SE)** | **Odds Ratio**  **(95% CI)** | **P-value** |
|  |  |  |  |
|  | Bedtime and sleep duration | | |
|  |  |  |  |
| Early Bedtime  (8:00 PM - 10:29 PM) | Reference | 1.00 (reference) |  |
|  |  |  |  |
| Regular Bedtime  (10:30 PM - 11:29 PM) | 0.57  (0.48) | 1.77  (0.7-4.5) | 0.23 |
|  |  |  |  |
| Late Bedtime  (11:30 PM - 1:59 AM) | 0.87  (0.66) | 2.38  (0.7-8.6) | 0.19 |
|  |  |  |  |
| Very Short Sleep Duration  (<7 hours) | **1.50**  **(0.72)** | **4.52**  **(1.1-18.5)** | **0.04*** |
|  |  |  |  |
| Short Sleep Duration  (7.0-7.49 hours) | **1.21**  **(0.62)** | **3.34**  **(1.0-11.2)** | **0.05*** |
|  |  |  |  |
| Conventional Sleep Duration  (7.5-7.9 hours) | Reference | 1.00 (reference) |  |
|  |  |  |  |
| Long Sleep Duration  (≥8.0 hours) | 0.62  (0.63) | 1.86  (0.5-6.4) | 0.32 |
|  |  |  |  |
| Calculations are based on binary logistic regressions modelling the association between sleep parameters and semen quality in relation to the reference group. Odds ratio: odds of reporting reduced semen quality. B: beta-coefficient, SE: standard error, 95% CI: 95% confidence interval. *significant at p<0.05. | | | |
